# The Consumption of Khat and Other Drugs in Somali Combatants: A Cross-Sectional Study

**DOI:** 10.1371/journal.pmed.0040341

**Published:** 2007-12-11

**Authors:** Michael Odenwald, Harald Hinkel, Elisabeth Schauer, Frank Neuner, Maggie Schauer, Thomas R Elbert, Brigitte Rockstroh

**Affiliations:** 1 University of Konstanz, Department of Psychology, Konstanz, Germany; 2 Deutsche Gesellschaft für Technische Zusammenarbeit, International Services, Addis Ababa, Ethiopia; 3 The World Bank Multi-Country Demobilization and Reintegration Program of the Greater Great Lakes Region, Goma, Democratic Republic of Congo; 4 Vivo International, Ancona, Italy; Campbelltown Campus University of Western Sydney, Australia

## Abstract

**Background:**

For more than a decade, most parts of Somalia have not been under the control of any type of government. This “failure of state” is complete in the central and southern regions and most apparent in Mogadishu, which had been for a long period in the hands of warlords deploying their private militias in a battle for resources. In contrast, the northern part of Somalia has had relatively stable control under regional administrations, which are, however, not internationally recognized. The present study provides information about drug abuse among active security personnel and militia with an emphasis on regional differences in relation to the lack of central governmental control—to our knowledge the first account on this topic.

**Methods and Findings:**

Trained local interviewers conducted a total of 8,723 interviews of armed personnel in seven convenience samples in different regions of Somalia; 587 (6.3%) respondents discontinued the interview and 12 (0.001%) were excluded for other reasons. We assessed basic sociodemographic information, self-reported khat use, and how respondents perceived the use of khat, cannabis (which includes both hashish and marijuana), psychoactive tablets (e.g., benzodiazepines), alcohol, solvents, and hemp seeds in their units. The cautious interpretation of our data suggest that sociodemographic characteristics and drug use among military personnel differ substantially between northern and southern/central Somalia. In total, 36.4% (99% confidence interval [CI] 19.3%–57.7%) of respondents reported khat use in the week before the interview, whereas in some regions of southern/central Somalia khat use, especially excessive use, was reported more frequently. Self-reported khat use differed substantially from the perceived use in units. According to the perception of respondents, the most frequent form of drug use is khat chewing (on average, 70.1% in previous week, 99% CI 63.6%–76.5%), followed by smoking cannabis (10.7%, 99% CI 0%–30.4%), ingesting psychoactive tablets (8.5%, 99% CI 0%–24.4%), drinking alcohol (5.3%, 99% CI 0%–13.8%), inhaling solvents (1.8%, 99% CI 0%–5.1%), and eating hemp seeds (0.6%, 99% CI 0%–2.0%). Perceived use of khat differs little between northern and southern Somalia, but perceived use of other drugs reaches alarmingly high levels in some regions of the south, especially related to smoking cannabis and using psychoactive tablets.

**Conclusions:**

Our data suggest that drug use has quantitatively and qualitatively changed over the course of conflicts in southern Somalia, as current patterns are in contrast to traditional use. Although future studies using random sampling methods need to confirm our results, we hypothesize that drug-related problems of armed staff and other vulnerable groups in southern Somalia has reached proportions formerly unknown to the country, especially as we believe that any biases in our data would lead to an underestimation of actual drug use. We recommend that future disarmament, demobilization, and reintegration (DDR) programs need to be prepared to deal with significant drug-related problems in Somalia.

## Abstract 2

Having interviewed military personnel in Somali, Michael Odenwald and colleagues conclude that drug-related problems, mainly relating to the use of khat, have reached proportions formerly unknown to the country.

## Editors' Summary

**Background.:**

Somalia—a country in eastern Africa—has been torn apart by civil war over the past few decades. Fighting among clans and warlords has caused the near-complete breakdown of state control in the central and southern regions of the country (including the capital, Mogadishu) although independent administrations provide some governmental control in the northern regions of Somaliland and Puntland. Efforts to establish a transitional federal government have largely failed and, to date, it has been impossible to initiate a nationwide disarmament, demobilization, and reintegration (DDR) program in Somalia for ex-combatants, a key step in the transition from war to peace. As in other war-torn countries, the social and economic reintegration of ex-combatants into civil society in Somalia is likely to be difficult. However, without effective reintegration, ex-combatants may take up arms again because they have no means of economic support or become disaffected and seek to destabilize the peace.

**Why Was This Study Done?:**

One risk factor for poor adjustment to civilian life among ex-combatants is substance abuse. Many ex-combatants use drugs to help them deal with traumatic war-related memories, but unrecognized drug abuse can hinder reintegration, increase criminality, and threaten the peace-building process. Most studies on substance abuse and treatment of drug-related problems of former combatants have been done in Western countries. Very little is known about how many ex-combatants abuse drugs and the types of drugs they abuse in postconflict regions in Africa. This information is needed if DDR programs are to be effective. In this study, therefore, the researchers have investigated drug use among “convenience” samples of combatants in seven regions of Somalia. Convenience samples are groups of people chosen to participate in a study because they were available rather than groups chosen randomly from the whole population.

**What Did the Researchers Do and Find?:**

Trained interviewers asked more than 8,000 military personnel about their own recent use of khat (chewing khat leaves releases an amphetamine-like stimulant), a legally traded drug in Somalia, where its use has long been commonplace. The interviewers also asked the respondents how much they thought others in their military personnel unit used khat and other drugs such as cannabis, psychoactive drugs (tranquilizers and other drugs that change mood, behavior, and thinking), solvents, alcohol, and hemp seeds. (Note that the researchers relied on perceived drug use; alcohol is illegal in Somalia, which is a Muslim country, and the use of drugs other than khat is not generally acknowledged.) Over the whole of Somalia, one-third of respondents said they had used khat recently. The highest levels of self-reported use were in southern/central Somalia, where up to two-thirds of combatants used it. More respondents in southern/central Somalia reported using an excessive amount of khat (more than two “bundles” of khat per day for one week) and having sleepless nights (a side-effect of khat) than in northern Somalia. The overall perceived use of khat (two-thirds of combatants) was higher than the self-reported use but similar in northern and southern/central regions. Finally, the perceived use of other drugs was highest in the southern/central regions.

**What Do These Findings Mean?:**

The use of convenience samples (which may not be representative of the whole population) and other aspects of this study mean that the numerical values of these findings may be inaccurate. For example, the levels of self-reported khat use may be underestimates because drug-using combatants may have been undersampled or not all combatants may have responded honestly. Nevertheless, these findings confirm that khat is the most commonly consumed drug among combatants and reveal a large increase in the number of people using it in southern/central Somalia since the conflict began (only one in five adult males used khat in these regions in 1980). They also reveal that more khat is being consumed by some individuals than previously, particularly in the southern/central regions, and uncover a worrying increase in the perceived use of other drugs, again mainly in the southern/central regions. These changes in the traditional patterns of drug use in Somalia, if confirmed in studies that use random sampling methods, suggest that future DDR programs in Somalia will need to be prepared to deal with major drug-related problems and that drug use among the general population might have reached dimensions formerly unknown to the country.

**Additional Information.:**

Please access these Web sites via the online version of this summary at http://dx.doi.org/10.1371/journal.pmed.0040341.

- Information about Somalia is available from the US Department of State, the United Nations, and Swiss Peace, a peace research institute
- The US Council on Foreign Relations, the Beyond Intractability Knowledge Base Project (based at the University of Colorado), and the UN DDR Resource Centre provide general information on DDR programs
- The Multi-Country Demobilization and Reintegration program provides additional information about ongoing DDR programs in other parts of Africa (in English and French)
- DrugScope (a UK charity) provides information about khat
- The US National Drug Intelligence Center provides information about khat in the US
- The UK Advisory Council on the Misuse of Drugs provides an assessment of the risk of khat to individuals and communities in the UK
- The Vivo Foundation supports programs that relieve the trauma of stress, including PTSD

## Introduction

Little is currently known about the prevalence of drug consumption by former combatants in states or countries that can no longer perform basic security and development functions, and that have lost effective control over their territory. Based on interviews of large numbers of combatants, we report on khat and other drug use among active armed forces and militia personnel in Somalia, where decades of civil war have produced a vacuum of state power [1] and where in some regions law is not enforced. The recently published Failed States Index 2007 ranks Somalia as number three worldwide (behind Sudan and Iraq) [2]. In order to understand the current political situation in Somalia, it is essential to realize that its society is clan-based, with six major clan-families and some minority groups. In early 1991, the autocratic rule of the former president Siad Barre, which had increasingly relied on his closely related subclans, was overthrown by several other clan-based armed groups, which split off later in rivalry over power and resources and produced the disordered situation that continues today [3]. Early on, the two northern regions, which roughly correspond to the former British protectorate Somaliland in the northwest, and some years later Puntland in the northeast, built up independent administrations and largely managed to restore stability with state-like governmental control. In 2003, at the time of our interviews, in the centre and south of the country the situation was much more complex and numerous factions had maintained the armed conflict for more than a decade, rejecting the authority of the Transitional National Government in Mogadishu. The Somali capital, Mogadishu, had been divided among various “warlords,” and their struggle for power had completely paralyzed the political and economic development of the country. In 2004, a round of peace talks in Mbaghati, Nairobi, led to the establishment of a Transitional Federal Government (TFG). Our assessment was initiated by these peace talks and was meant to prepare the disarmament, demobilization, and reintegration (DDR) program in Somalia. However, after our interviews took place, the political division within the TFG made it impossible to start a DDR program, and in 2006 a completely new situation emerged: the United Islamic Courts (UIC) seized the capital, driving away the warlords who held positions as ministers and threatened the whole TFG. As a consequence, Ethiopia started a military intervention with the backing of Western governments to enable the TFG to gain control over Mogadishu. Since then, however, the country has not been stable (for a more detailed report of the current political situation in Somalia see [4,5]). Thus, the current political situation offers little hope for the effective re-establishment of a central government and an end to the armed conflict.

After the end of armed conflict, former refugees, internally displaced people, and ex-combatants must be socially and economically reintegrated. During this process in many countries, ex-combatants were frequently found to have severe adjustment difficulties, e.g., in the form of occupational or marital problems [6–8]. Substance abuse and dependence have been identified as risk factors for different aspects of their readjustment to civilian life [9–12]. In particular, ex-combatants who had been exposed to ongoing traumatic stress, which may have resulted in post-traumatic stress disorder (PTSD), were frequently found to have high levels of alcohol consumption as well as the use of other substances [13–16]. Current knowledge supports the self-medication hypothesis, i.e., drugs are consumed to suppress war-related traumatic memories, improve sleep, or ameliorate depressive symptoms [17–20]. Thus, in recent years psychiatric services for ex-combatants developed more integrated models of care, including the treatment of substance abuse [21,22]. However, most studies on substance use, readjustment, and treatment of drug-related problems of former combatants have been conducted in Western countries. Information from African postconflict regions are sparse, especially related to the prevalence of abuse, the type of drugs consumed, and the relation of drug use to other psychiatric conditions and readjustment measures. This lack of information hinders the efforts made by reintegration programs in many resource-poor postconflict countries, as unrecognized drug abuse might lead to failure of reintegration in a substantial number of cases, which, in turn, could lead to social problems (e.g., increased criminality [23]) or lower returns for the whole national economy. Widespread drug abuse might even affect the peace-building process as a whole [24], e.g., when many ex-combatants turn to drug trafficking or banditry, as some examples show [25].

The main drug abused in Somalia is khat [26]. The leaves of the khat shrub are traditionally consumed in parts of Africa and in Arab countries for their stimulating properties [27,28]. Recently, as khat is used by immigrant groups, its use has spread to Western countries [29,30]. The chemically unstable alkaloid cathinone, S(-)alpha-aminopropiophenone, present in the fresh plant material, is the main psychoactive agent [31]. Numerous laboratory studies confirmed that cathinone resembles amphetamine in chemical structure and affects the central and peripheral nervous system [32] and behavior [33,34] similarly (for a review see [35,36]). Khat use has been related to numerous somatic and psychiatric health sequelae [37–39].

The information currently available concerning drug intake in Somalia is sparse. In the last cross-sectional assessment of khat intake before the collapse of state, Elmi [40] reported that the khat habit was introduced to the southern part of the country only after 1960, in contrast to the north, where its use is a long-standing tradition; his assessment in the 1980s revealed that in the north of the country, 64% of adult males from the general population regularly consumed khat compared to 21% in the south. More recent data from the neighboring countries indicate that khat chewing is frequently comorbid with alcohol consumption, smoking cannabis (which includes both hashish and marijuana), and intake of benzodiazepines [41–43]. We recently reported a cross-sectional study in northwestern Somalia (Somaliland) [44] showing that self-reported khat use was more frequent and excessive among male ex-combatants (60%) than among adult male civilian war survivors (28%) and males without war experience (18%; *p* < 0.001). In recent years, there is growing evidence that khat-related business is one source of income for civil war factions in southern Somalia [45] and that the cultivation and trafficking of cannabis and other illicit drugs constitutes an increasing problem, but available data have remained limited [46]. In all of Somalia, drug control and rehabilitation efforts are not exceeding primary levels: khat is legally traded and little is done to raise public awareness of its potential dangers. Alcohol is illegal because of religious beliefs; other drugs are not officially acknowledged as a problem. This lack of acknowledgment must be understood in context: the former, Siad Barre regime had tried to ban khat, and its trade and import tax are main sources of income for civil war factions in the south as well as for the regional administrations in the north. Nevertheless, various local nongovernmental organizations have started to address khat-related health problems as shown by many hand-painted billboards in Somali towns promoting “khat counseling” together with other psychosocial services such as “HIV counseling” (for a detailed report see [47]). In the area of DDR, the international agencies have only just started to address the drug problem of ex-combatants in Somalia [48], but so far no systematic approach has been worked out.

Based on seven large convenience samples from different regions of Somalia, we present data based on self-reported khat use among active security personnel and militia in Somalia, some problematic use patterns (e.g., solitary and excessive use), and perceptions among the participants about the prevalence of the intake of six types of narcotic drugs in their units. We were especially interested to estimate how many of the potential future participants of demobilization and reintegration activities use khat, which drugs are the most frequently consumed according to the perception of respondents, and whether there are differences between northern Somalia and the southern/central part of the country, where a larger increase of drug-related problems can be expected.

## Methods

### Design, Sampling, and Participants

The exact number of armed staff and militia personnel in Somalia is not known. In 2003, international organizations estimated that the total number lies between 70,000 and 80,000 men under arms [49]. Of them 17,600 were in Somaliland, 6,500 in Puntland [50], and approximately 17,000 in Mogadishu [49]. In addition, we used the proposed numbers of demobilization camps to estimate militia numbers in other regions (see Table 1).

**Table 1:**
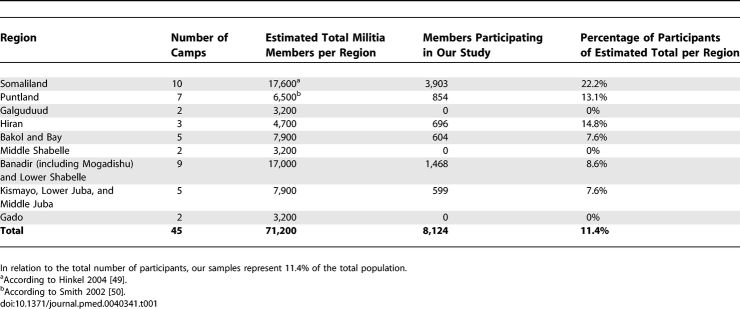
Estimates of Total Number of Militia Members in Different Regions of Somalia Based on Estimates for the Construction of Demobilization Camps in 2003 [49]

Given that the country remains in armed conflict and sampling of armed staff of opposing factions was based on vague political arrangements, it was not possible to apply random sampling methods as we initially intended, because necessary information about group size, location, and so forth was, understandably, not revealed. Thus, we drew seven large convenience samples in seven parts of the country. The use of convenience samples does not allow for the estimation of biases, and thus results must be interpreted with caution. However, under certain conditions the use of convenience samples in studies determining prevalence of drug use is justified [51], and especially in clinical research, studies with large convenience samples actually have produced estimates that come very close to those of studies using more rigorous sampling strategies [52–54].

The seven parts of Somalia with the highest estimated military staff density were selected for interviews (see Table 1), including major population centers and rural areas: In the north of Somalia, we included Somaliland and Puntland (both regions have governing regional administrations); in central Somalia, Hiran; and in southern Somalia Bay/Bakol, Mogadishu and Kismayo (this division is used as convention, for the comparisons between northern and southern/central Somalia mentioned below). Bay and Bakol are two regions dominated by the Rahaweyne clan. Kismayo included parts of the Lower and Middle Juba regions; at the time of the interviews, this region was dominated by the Juba Valley Alliance, which tried to establish an independent administration as in the northern regions. Mogadishu was divided in two samples according to the “green line,” which divided the town between main factions at the time of the interviews, labeled “Mogadishu North” and “Mogadishu South.” Mogadishu South included the Lower Shabelle region with the town of Merka. In central and southern Somalia, the territory was divided between a large number of smaller and bigger factions, who, at that time, did not allow a central government to exert power [3]. Interviews were conducted with all collaborating groups, asking for a minimum of one unit within the overall structure to be completely assessed. We had no access to basic information concerning the units (e.g., actual size of units) chosen for this assessment. In southern/central Somalia, of the major factions, all but one collaborated with this research project. In Somaliland and Puntland, due to the previous establishment of regional administrations, regional armed forces had been established by incorporating the former clan-based factions. In Somaliland, the project made use of the ongoing DDR activities, which included registering armed staff on the government payroll. The study used all data available until the project had ended. In Puntland, we interviewed military units under the regional administration in three major population centers and one other armed group. In every region, we aimed to assess a minimum of 600 respondents, including as many factions and armed groups as possible.

Interviews were conducted between August and December 2003. The interviewers went directly into a compound used by the respective militia or units to conduct the individual interviews in a place that provided as much privacy as possible, e.g., in a separate room. As participation in the assessment was ordered by superiors, we may assume a nearly 100% participation rate initially. But before asking for informed consent in the individual interview setting, all participants were assured that a refusal to participate would not be reported to superiors. According to the observation of interviewers, the motivation of the individual unit members to participate in the assessment was very high. Despite the fact that they were accurately informed about the study, and that their participation would not have any effect on the participation in a future program, they still hoped that they might increase their chances of being selected by participating in the assessment.

In total, 8,723 militiamen and security staff were interviewed; 587 of them were excluded from the analysis because they denied their consent after being informed about the purpose of the study (empty sheet returned) or during the interview (6.7%) and 12 because their interviewers did not fulfill minimal standards (a minimum of ten interviews per interviewer was required), resulting in 8,124 (93.1%) interviews entering statistical analysis. We reached 22% of armed staff in Somaliland and about 11% of the total estimated number of armed personnel in all Somalia. Of them, 4,070 belonged to regional authorities and 2,290 to warlord factions, 1,090 were members of freelance and clan-based militia, 481 of sharia court militias, and 78 members of business militias.

### Interviewers, Training, and Supervision

Interviewers were staff of local nongovernmental organizations (NGOs) with interviewing experience (*n* = 38). Prior to data collection, a 14-d training was conducted that contained clinical concepts and research design, role playing, and field exercises. Contact with interviewers was maintained throughout the assessment phase by cell phone, radio, and field visits by a team member of Somali origin and international staff. Close contact with the forces' and militias' command and a large-scale awareness campaign by local press, radio, and TV prepared the ground. There were no security incidents reported during the assessment. After the assessment, local NGOs handed in the questionnaires and assisted data entry.

### Instrument

The questionnaire was designed as a standardized instrument of relevant individual information of active militiamen and security personnel in order to prepare for DDR activities. Questions and closed answers were developed in English by an interdisciplinary team consisting of Somali medical and nonmedical staff and international experts on demobilization and mental health staff from various organizations. Within this group, all items were extensively discussed to determine cultural appropriateness, translated, and, then independently retranslated by professional translators. If the back translation revealed a discrepancy in semantic content, the item was revised and again, independently retranslated. The back-translation process was repeated as often as necessary until the semantic content of the Somali wording was adequate.

The results of the sociodemographic part of the interview are presented in Table 1.

Types of narcotic substances to be assessed were defined in a prior assessment among the 45 participants of the interview training (some of them did not become interviewers later). As a result, the following six types of narcotic drug consumption were assessed in the main study: (a) chewing khat, (b) smoking “cannabis,” (c) ingesting psychoactive “tablets” (not prescribed but taken in order to be “high,” e.g., diazepam pills), (d) drinking alcohol, (e) inhaling solvents (e.g., petrol or glue), and (f) eating “bangi” seeds (hemp seeds). Since, in the interviewers' opinion, khat was the most widely used drug, we assessed for self-reported khat use and the consumed quantity during the week before the interview. Self-report of individual drug intake was found to be a reliable and valid measure in Western countries [55], which can be related to other risky behaviors [56]. In order to facilitate honest answers, we first asked respondents what type of khat they would usually use, the Ethiopian type (“Herari”) or Kenyan type (“Miraa”): “What kind of khat do you usually chew?” Then we asked for the number of traded units “ready to consume” (“bundles”, “kilos”, “mijins” or “marduufs”, which include approximately 100–300 g of the leaves [57], 100 g of fresh leaves from different origin contain on average 78–343 mg of cathinone [58]; one study reported a cathinone content of 1,140 mg/100 g in one sample of leaves [59]) consumed in the previous week: “How many bundles of khat have you consumed in the last week?” We determined an average consumption of more than two bundles per day to be “excessive,” as this margin was found to be the threshold that separated khat users with psychiatric problems from those who were psychologically healthy [60]. We also inquired about typical effects associated with excessive chewing such as: (a) sleepless nights due to continued khat intake, which occurs frequently in heavy chewers because of its stimulating effects and (b) consumption in a solitary setting, which was described as a habit of “problem chewers” [61] which is in contrast to the traditional “social” setting (khat party).

In some parts of the country, the consumption of drugs such as alcohol can involve severe punishment; therefore, we assessed for the consumption of all other types of drugs by asking for perception of their use. First, we assessed the respondents' opinion about the existence of the six types of drugs defined above: “Are you aware of the following drug-taking habits in your locality?” Then, we asked them to estimate the percentage of his/her unit members who had consumed these drugs in the week before the interview (perception of use): “In your opinion, what percentage of people in your (force/militia) unit have used the following drugs in the last week?” In western samples, the perception of drug use of peers has a high predictive value for the self-reported drug use [62]. The validity of the items concerning sociodemographic information and self-reported khat use was demonstrated in our previous work [44]. Using the same methodology and procedure in a previous study with ex-combatants [63], the same local interviewers who assisted in the current study had assessed quantitative information on khat use. A second, clinical interview by trained international experts with the help of trained interpreters was conducted in 64 subjects, about one to four weeks later, in order to evaluate the first interview. Given that both interviews assessed drug use information with reference to different weeks, the correlation between the quantitative information on khat use was high (*r* = 0.69, *p* < 0.001) and the correspondence of the categorical information was satisfactory (kappa = 0.47, *p* = 0.001).

### Approval and Ethics

The Cease Fire, Disarmament, and Demobilization Committee (Committee 2) of the Somali Peace and Reconciliation Conference in Mbaghati, Nairobi, the National Demobilization Committee in Hargeisa, the Somalia Unit of the European Commission (Nairobi), the German Agency for Technical Cooperation, International Services (GTZ IS; Nairobi) approved the assessment. All participants were informed before the interview about purpose and method of the interview, about confidentiality and about the possibility to discontinue the interview at any time without negative consequences. Interviews were only conducted after respondents had given verbal consent. We accepted oral consent because of the high rate of illiteracy.

### Statistical Analysis

Data were analyzed with SPSS 11.0 for Macintosh and SPSS 15.0 for Windows. With respect to the different regions, we report proportions in percents and for continuous variables, means and standard deviations (SDs). For all point estimates, we report 99% confidence intervals (CIs). As approximate statistics for the total sample and for northern and southern/central Somalia we calculated weighted means, standard errors (SEs), and proportions as well as their 99% CIs. For this analysis we treated the data as if they were from a one-stage random cluster sampling, with same inclusion probability on the level of regions (clusters) and weights for individual data based on inclusion probabilities in the different regions (number sampled per region/total per region). Because of the interpretation problems of data from convenience samples, we wanted to focus on the clearest differences and opted for Alpha 0.01. In order to describe the co-occurrence of different drugs in the same military units, we used Pearson's *r* for the estimated proportions of drug users for the total sample. We do not report statistical tests and *p* values, as this is problematic with our sampling method and as they are not informative with our very high *n* (e.g., for *r* > 0.04 *p* < 0.001). Differences between regions are discussed based on confidence intervals.

## Results

### Sociodemographics

In our sample we found that 89.1% of respondents were male (99% CI 70.4–96.6), this proportion tends to be larger in the northern part of the country (see Table 2). The average age of respondents was 37.3 y (99% CI 29.4–45.2). Although the age difference between northern and southern/central Somalia was 8 y, it did not reach statistical significance. The oldest respondents were found in Somaliland (41.3 y, 99% CI 40.8–41.9 y) and Hiran (39.6 y, 99% CI 38.4–40.8 y). However, in the three samples from the regions with highest levels of violence, the mean age was approximately 30 y, clearly younger than in all other regions (Mogadishu North 29.1 y, 99% CI 28.1–30.0 y; Mogadishu South 30.3 y, 99% CI 29.5–31.1 y; Bay/Bakol 30.5 y, 99% CI 29.6–31.5 y). Our respondents from the northern part of the country were more frequently married (north: 74.1%, 99% CI 71.4%–76.6%; southern/central Somalia: 58.3%, 99% CI 44.7%–70.7%), but the number of their dependents did not seem to differ. In the whole sample, 32.3% completed their primary education (99% CI 26.1%–39.2%) and 23.4% had received any vocational training (99% CI 14.2%–36.1%); no differences were found between the two parts of the country, but the regional coefficients differed substantially. In relation to the war history of respondents, we found that on average 65.1% had combat exposure (99% CI 30.0%–89.0%). This percentage did not differ significantly between northern and southern/central Somalia (54.3%, 99% CI 30.5%–76.3% versus 80.2%, 99% CI 36.7%–96.6%); but in the Somaliland and Kismayo samples this proportion was less than 50%, lower than in all other regions. The self-reported age of first combat exposure was 22.9 y (99% CI 19.7–26.0 y) and did not differ between northern and southern central Somalia (24.5 y, 99% CI 23.1–25.9 y versus 21.4 y, 99% CI 18.6–24.2 y); but again we found the youngest combatants in the three regions with highest levels of violence (Mogadishu North 20.0 y, 99% CI 19.0–20.9 y; Mogadishu South 19.8 y, 99% CI 19.3–20.4 y; Bay/Bakol 21.6 y, 99% CI 20.9–22.3 y). The self-reported duration of combat exposure was 1.9 y in the whole sample (99% CI 0.9–2.9 y) and tended to be larger in the northern regions (2.5 y, 99% CI 2.1–2.8 y versus 1.4 y, 99% CI 0.6–2.1 y).

**Table 2:**
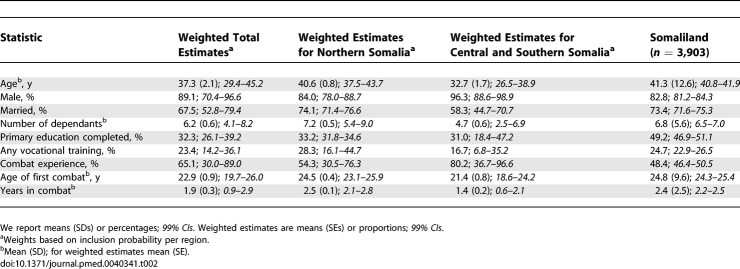
Sociodemographic Information on 8,124 Active Armed Forces and Militia Staff in Seven Regions of Somalia (Continued on Next Page)

**Table 2:**
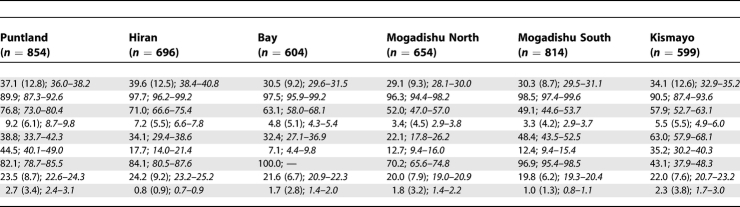
Extended.

### Self-Reported Khat Use

**Figure 1:**
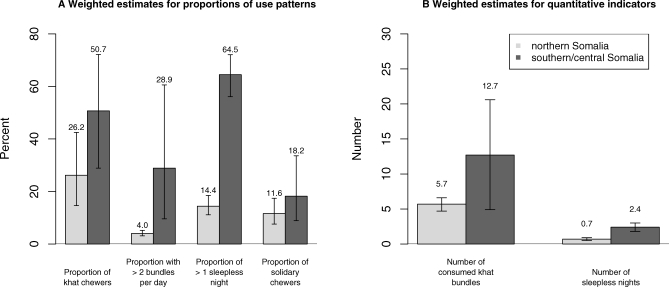
Comparison of Information on Self-Report Information on Drug Use of 4,751 Respondents in Northern and 3,373 in Southern/Central Somalia (A) Weighted estimates of proportions of khat users (week before the interview) and of respondents reporting signs of excessive use (> 2 bundles/d, > 1 sleepless night/wk, chewing alone); weights are based on sampling probability per region; error bars correspond to the 99% CIs. (B) Weighted estimates of average quantity of khat (bundles) consumed by 1,248 users in the north and 1,707 in the south and number of sleepless nights due to khat chewing in the week prior to the interview; weights are based on sampling probability per region; error bars correspond to 99% CIs.

In all Somalia, 36.4% of respondents reported khat intake in the week previous to the interview (99% CI 19.3%–57.7%; see Table 3), with the highest levels of all in Bay/Bakol and Mogadishu South (69.4%, 99% CI 64.5%–74.2% and 61.4%, 99% CI 57.0%–65.8%). The estimates for the northern and southern/central part of the country did not differ (26.2%, 99% CI 14.6%–42.5% versus 50.7%, 99% CI 28.9%–72.2%). However, excessive khat use and its related effects seem to be more frequent in southern/central Somalia: consumption of more than two “bundles” per day in the previous week was less frequent in the northern part than in southern/central Somalia (4.0%, 99% CI 3.1%–5.2% versus 28.9%, 99% CI 9.6%–60.9%). Also in this region a greater number of respondents reported more than one sleepless night due to khat chewing in the week prior to the interview (14.5%, 99% CI 11.1–18.5 versus 64.5%, 99% CI 56.1–72.1); but habitual consumption in a solitary setting was not significantly different between regions (11.6%, 99% CI 7.6%–17.4% versus 18.2%, 99% CI 8.9%–33.6%). The self-reported, quantitative information about individual khat chewing in the week before the interview follows the same trend (see Table 4): khat chewing respondents reported that they consumed on average 9.8 “bundles” (SE = 2.1, 99% CI 2.0–17.5) and had 1.7 sleepless nights due to chewing khat in the previous week (SE = 0.4, 99% CI 0.1–3.3). Again, the differences in the reported number of sleepless nights due to khat use in the previous week between northern and southern/central Somalia were evident (0.7, 99% CI 0.6–0.9 versus 2.4, 99% CI 1.8–3.0) but not significant with respect to the consumed quantity (5.7, 99% CI 4.7–6.6 versus 12.7, 99% CI 4.9–20.6). On a regional level, we found the largest consumed quantities (expressed in bundles) in the samples in Mogadishu South (including Lower Shabelle and the “Km 50 Airport,” where many khat-delivering planes land daily: 15.9, 99% CI 14.4–17.5) and in Kismayo (including Lower and Middle Juba; 20.8, 99% CI 18.0–23.6), the two regions closest to the Kenyan border, from where the largest quantity of khat imports arrive. Figure 1 displays graphically the differences between the north and central/southern Somalia.

**Table 3:**
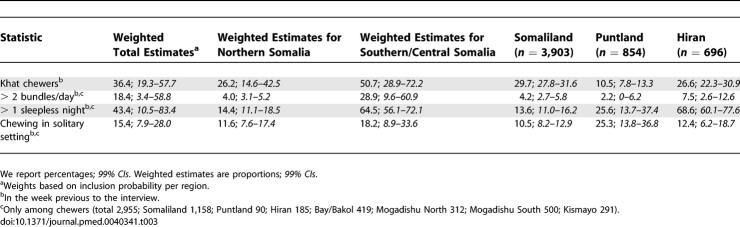
Qualitative Information about Chewing Khat and Associated Features Based on Self-Report in Active Armed Forces and Militia Staff in Seven Regions of Somalia (Continued on Next Page)

**Table 3:**
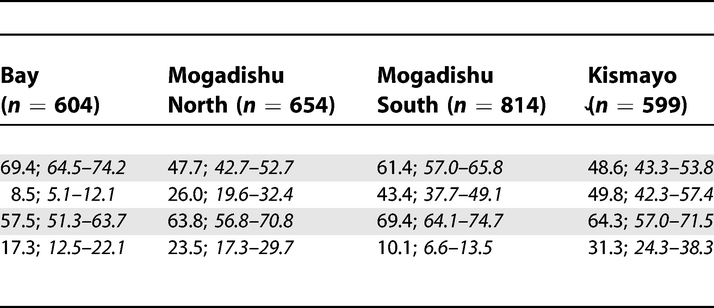
Extended.

**Table 4:**
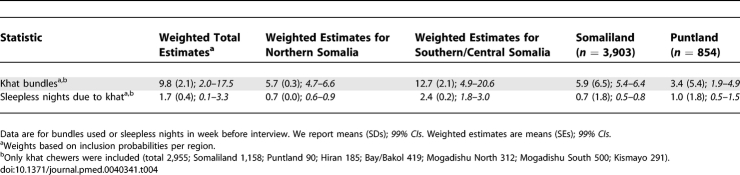
Quantitative Information Based on Self-Report Assessment Given by Active Armed Forces and Militia Staff about Their Khat Consumption in the Week Previous to the Interview in Seven Regions of Somalia (Continued on Next Page)

**Table 4:**
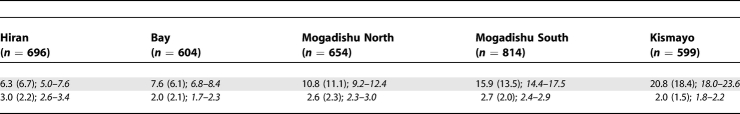
Extended.

### Perceived Drug Use in Locality and in Military Units

Table 5 displays the Somali militia/security personnel's perception of the existence of different types of drug consumption. Based on our estimates, 96.5% (99% CI 92.5%–98.4%) of respondents believe that khat is consumed in their locality, without much variation between regions. Smoking cannabis is the second most frequently perceived type of drug use: 40.4% (99% CI 8.0%–81.4%) of respondents believe that it exists in their localities, followed by intake of psychoactive tablets (34.9%, 99% CI 5.6%–82.9%), drinking alcohol (30.6%, 99% CI 10.2%–63.1%), sniffing inhalants (16.9%, 99% CI 4.4%–47.5%), and eating bangi seeds (7.6%, 99% CI 0.8%–46.8%). There are marked differences on the regional level about the perceived consumption of cannabis (north: 15.7%, 99% CI 13.7%–18.1% versus southern/central: 75.2%, 99% CI 51.9%–89.5%), psychoactive tablets (10.5%, 99% CI 6.2%–17.3% versus 69.1%, 99% CI 45.2%–85.8%), and alcohol (17.1, 99% CI 16.7%–17.4% versus 49.7, 99% CI 23.4%–75.2%).

**Table 5:**
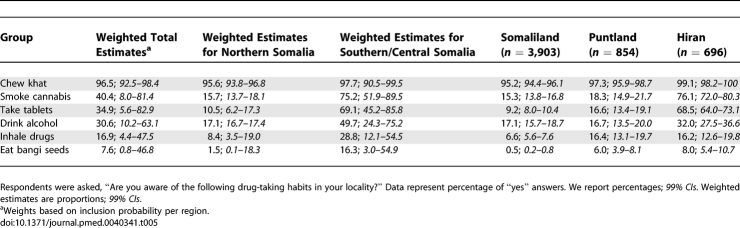
Respondents Who Believe That the Respective Drug Is Consumed in Their Localities, Reported as Proportions of “Yes” Answers in the Seven Regions and Corrected Total Proportions (Continued on Next Page)

**Table 5:**
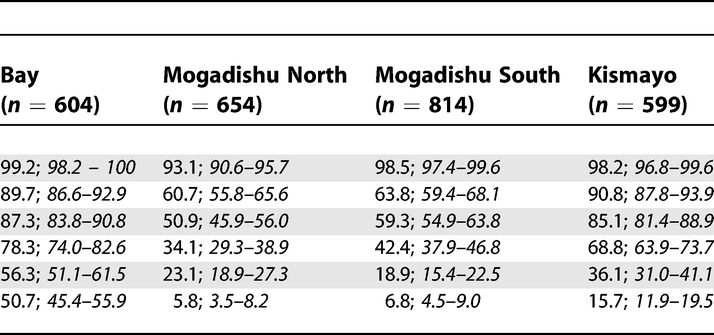
Extended.

**Figure 2:**
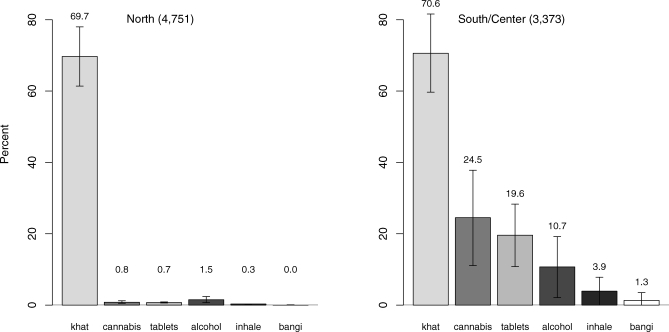
Respondents' Perceptions of How Many of Their Unit Members Consumed Six Types of Drugs in the Past Week (Percentages), in Northern Somalia (Somaliland and Puntland) and Central and Southern Somalia (Hiran, Bay, Mogadishu, and Kismayo) We report weighted estimates for proportions; weights are based on sampling probability per region; error bars correspond to the 99% CIs.

The estimated percentages of drug consumers among armed personnel (see Table 6) showed the same patterns. Respondents thought that on average 70.1% (99% CI 63.3%–76.5%) of their comrades chewed khat in the week before the interview. In this respect, again, there is no difference between north and south (69.7%, 99% CI 61.4%–78.0% versus 70.6%, 99% CI 59.7%–81.6%). Based on our estimates, respondents believed that cannabis was consumed by 10.7% (99% CI 0%–30.4%), tablets by 8.5% (99% CI 0%–24.4%), alcohol by 5.3% (99% CI 0%–13.8%), inhalants by 1.8% (99% CI 0%–5.1%) and bangi seeds by 0.6% (99% CI 0%–2.0%) in the previous week. In southern/central Somalia the estimated proportion of users was higher than in the northern part of the country with respect to cannabis (north: 0.8%, 99% CI 0.5%–1.2% versus south: 24.5, 99% CI 11.1%–37.8%) and psychoactive tablets (0.7%, 99% CI 0.6%–0.9% versus 19.6, 99% CI 10.8%–28.3%); no differences were found for alcohol (1.5%, 99% CI 0.7%–2.4% versus 10.7%, 99% CI 2.1%–19.2%), inhalants (0.3%, 99% CI 0.2%–0.3% versus 3.9%, 99% CI 0%–3.9%) and bangi seeds (0.0%, 99% CI 0%–0.1% versus 1.3%, 99% CI 0%–3.5%). On a regional level, we found the highest levels of cannabis and tablet use in Bay/Bakol (38.1%, 99% CI 35.6%–40.6%; 30.2%, 99% CI 27.7%–32.7%) and Kismayo/Lower and Middle Juba (33.2%, 99% CI 30.4%–35.9%; 22.6%, 99% CI 20.4%–24.8%). Figure 2 reports these differences between northern Somalia and the southern/central part of the country.

**Table 6:**
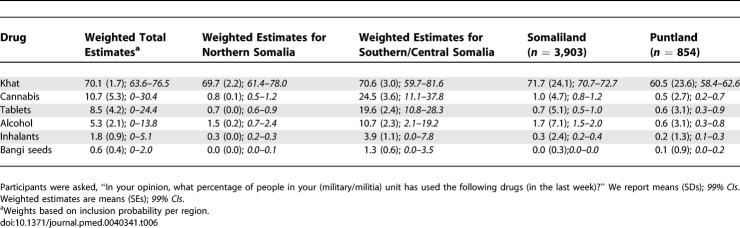
Estimated Percentages of Consumers of Six Types of Drugs in Armed Forces and Militia Units in Somalia (Continued on Next Page)

**Table 6:**
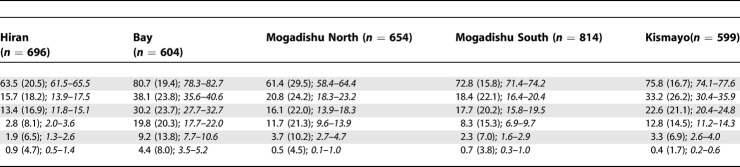
Extended.

### Correlation between Perceived Use of Different Types of Drugs in Military Units

We calculated the correlations between the perceived proportions of unit members believed to take the different types of drugs (see Table 7). The perceived proportion of khat consumers is not strongly associated with any of the other drugs (0.08 ≤ *r* ≤ 0.22). However, the perceived proportion of cannabis consumers substantially correlates with those of tablet consumers (*r* = 0.76) and alcohol drinkers (*r* = 0.53). The perceived proportion of alcohol drinkers correlates with that of tablet users (*r* = 0.56) and inhalers (*r* = 0.50). The perceived proportion of bangi seed consumers is associated with that of inhalers (*r* = 0.54).

**Table 7:**
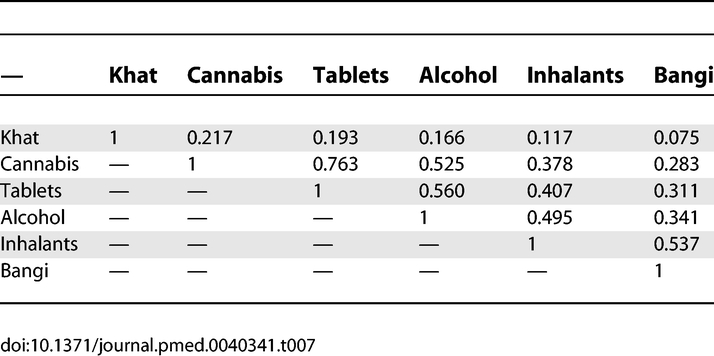
Correlations between Estimated Percentage of Use of Six Different Types of Drugs in Military Units

## Discussion

The exact numeric results of our assessment of 8,124 active armed forces and militia personnel in Somalia must be interpreted with caution, because the sampling methods that were used would not allow for estimation of biases. We found that sociodemographic characteristics of combatants differed according to the level of conflict in the respective region. In the two northern samples, Somaliland and Puntland, and the most southern one (Kismayo and Lower and Middle Juba), where one single administration was in place in the years preceding the interviews and where outbreaks of violence were locally and temporarily restricted, respondents seemed to be older, more often married, and better educated. The proportion of female respondents also was higher compared to the regions with “hot conflicts”, too—a phenomenon probably related to the Somali tradition that family members inherit a paid position of a deceased person, which probably cannot be maintained under conditions of ongoing armed conflict.

Our assessment revealed that khat chewing is very frequent; while the self-reported numbers reach up to nearly 70% in the regional sample of Bay/Bakol, we found an overall estimate of 36%. According to the perception of the participants, khat is the psychoactive drug most often consumed, and estimated levels of its use are similar in all samples, reaching 70%. As for western settings, data on self-reported drug consumption and perceived consumption do not match [62]. The discrepancy that we observed in our Somali samples is similar, and reflects that self-report seems to be an underestimate, and perceived consumption by peers an overestimate, of real drug use figures. The assessed behaviors associated with problematic khat chewing show that severe addiction must be expected in a substantial part of the samples: excessive use, as we defined it here, (on average more than two bundles a day in one week) was reported by 18%, more than one sleepless night due to khat chewing in the last week by 43%, and solitary consumption by 15%. Other drugs, formerly rarely used in Somalia, are also consumed, especially cannabis, psychoactive pills (such as benzodiazepines), and alcohol. The regional differences of drug intake in our sample are remarkable: in southern Somalia we found a higher self-reported excessive khat intake and a unanimous perception of higher consumption levels of cannabis and psychoactive tablets.

Irrespective of the numerical details, general conclusions can be drawn from these findings: First, today khat is the most consumed drug among military staff and probably also among the general population in all parts of Somalia. In this respect, our results here are in complete agreement with previous reports [40]. Moreover, this view is supported by the sparse but impressive numbers that exist on khat importation to Somalia: In 2000–2001, 12,100 metric tons of officially imported khat with a value of US$37.5 million found their way from Ethiopia to Somalia [47]. Estimates of imported khat shipments from Kenya range up to US$300,000 per day, which would make more than US$100 million per year [47].

Second, according to the perception of participants, the percentage of khat users among armed personnel of southern Somalia equals that of the northern part. This observation is in contrast to the last epidemiological study on khat use [40], which found higher levels in the north, but which studied only the general population. Based on the assumption that this result was indicative of the overall prevalence of khat use, we speculate that khat use among vulnerable groups such as militia, and probably also among the general population, has dramatically increased in southern Somalia during the course of the civil war.

Third, in our study we find evidence that drug use in Somalia has not just changed quantitatively but also qualitatively: data on perceived drug use documents the consumption of traditionally unknown and formerly unaccepted substances, such as benzodiazepines or alcohol. Our data also imply that the formerly unknown poly-drug use seems to exist in militia units; according to the perception of our respondents, in places where bangi seeds are consumed, the probability is above 86% that all other drugs are consumed, too. Unsystematic observations during our field work in Somalia and published studies from neighboring Ethiopia showing the combined use of khat and other drugs [41] supports this hypothesis. Although we know that the perceived peer alcohol use contains a bias toward overestimation [62,64,65] and that our findings need to be replicated by studies relying on self-report, clinical interviews, and objective test methods in representative samples, poly-drug use seems to be a new development despite the importance of Islam in this country. Further, our data strongly support the hypothesis that in Somalia, a subgroup of individuals show excessive (khat/drug) abuse patterns and severe impairment in everyday functioning; these people may suffer from severe forms of drug dependence and related psychiatric problems [66,67]. This finding parallels some studies among Somali immigrants in the UK where a proportion of the respondents showed severe forms of khat addiction and comorbid abuse of other drugs [68,69]. Thus, it is plausible to assume that these severely addicted, multiple drug users are not solely found among military personnel but also in other vulnerable groups. Our data suggest that qualitative alterations in drug usage are more pronounced in southern Somalia. This notion would imply potential threats for the peace-building process in general (e.g., drug-related criminality) and the reintegration of former combatants into civilian society in particular [9,10].

In sum, we argue that drug use, which was traditionally considered a problem of the north of the country, has increased to alarming levels among military personnel and other vulnerable groups, especially in the southern part of the country. Based on the scarce information about the widespread availability of drugs, which documents an increased drug production and trafficking in southern Somalia [46], and in combination with high levels of unemployment, especially among the young, as well as the overall substance use trend in sub-Saharan Africa [25], it is likely that drug abuse in southern Somalia could also increase within the general population. Unfortunately, there are currently no data available that would allow a firm statement on this issue.

There are several reasons that would explain why drug use among militia in southern and central Somalia is higher than in the north. The different levels of governmental power and law enforcement are one obvious explanation, supporting the notion that the collapsed state power might increase levels of drug abuse, particularly in postconflict or traumatized societies. A weak, ineffective, or nonexistent government might facilitate the unrestrained production, importation, distribution, and consumption of drugs, as seems the case in southern and central Somalia. In the militia groups, drugs like cannabis and benzodiazepines might be easily available and disciplinary consequences missing. We propose that three main factors facilitate the use of khat by Somali militia members in southern and central Somalia: the proximity to Kenyan khat production areas, where trade is in the hands of Somali middlemen; the known involvement of factions in the khat business as a means of income [45]; and the control of airstrips and roads by war-lords (who might charge tolls in the form of a share of the transported khat) . Some even speculate, based on field observations and anecdotal reports, that in some militia groups khat forms part of the salary [45]. Additionally, there are concerns that in the fertile regions of southern Somalia, notably the valleys of the Shabelle and Juba rivers, the cultivation of cannabis is increasing [46], leading to a higher availability in the local markets. This reasoning is supported by our finding that in Kismayo, the economic center next to the lower Juba valley, and the neighboring regions of Bay and Bakol, levels of perceived cannabis use are highest. Furthermore, drug use in the unstable southern and central regions might be influenced by the ongoing armed conflict, in the sense that militiamen, who had been exposed to a high degree of violence, might use drugs as self-medication in order to cope with the traumatic experiences—a result that is in line with studies on increased drug use by Western ex-combatants who are either affected by PTSD [17] or were exposed to high levels of war-zone stress [13].

During the short period of the UIC regime, khat use was decreased [70], but this did not induce long-lasting changes. According to local informants, a day after the UIC was expelled from Mogadishu, the khat business continued as if there had been no interruption.

Our data raise some important methodological questions concerning the assessment of drug use in Somalia. According to our experiences in a preparatory study [63], answer tendencies play a major role in the self-reported khat use. The wording of the questions has a marked effect on the respective answers; for instance, when asked directly whether a respondent would chew khat, almost 100% of respondents answered “no,” even though this was obviously in strong contrast to our daily observation of the respondents. An indirect style of questioning, such as employed in this study, however, produced answers with more face validity. Still, we believe that this method of self-report produces underestimates of real figures. We observed that many respondents answered that they had stopped chewing khat one week or one month before and thus failed to report khat use in the week preceding the interview. Many respondents obviously had a strong wish to discontinue their own khat consumption, which was contrasted by frequent relapses. On the other hand, we cannot rule out that social desirability played an important role in under-reporting, especially because of the respondents' fear that they would be denied access to DDR programs if they admitted their drug use. In particular, self-reported khat use might have been affected by under-reporting or nonreporting due to social desirability, in contrast to the perceived use by unit members. In contrast, one might argue that oversampling of drug-using individuals in southern and central Somalia might have occurred. However, our strategy of interviewing one complete unit may have circumvented such effects. This possibility is strengthened by the correspondence of both coefficients on khat use, based on self-report and on the perceived consumption within the units. Both showed a small to moderate north–south difference in the same direction, whereas only the self-reported drug use would be effected by oversampling of drug-consuming individuals in the south. In general, the prevalence rate of 36% self-reported khat intake might represent an underestimation of real numbers. In a previous study in Somaliland, 60% of ex-combatants reported that they had used khat in the week before the interview [44].

Our study has limitations. Because of the political preconditions of the assessment, which did not allow for random sampling, we had to work with seven unrelated convenience samples. Furthermore, because of the information control policy of collaborating factions, there is no complete list of militia units, and we do not know the actual number of armed personnel in the included units. This deficiency hampers the interpretation of the derived coefficients and prevents the estimation of the selection bias. Because participation was ordered by superiors, we might have included participants who, despite being informed that denial of their consent would not have any consequences, might have decided to give incorrect answers. However, the fact that hundreds of respondents confirmed that alcohol intake (against Islamic law) exists in their community suggests that answers were often honest. Furthermore, the low rate of dropout after being provided with information about the study and during the course of the interview (6.3%), the large size, the inclusion of all factions but one in important regions of the country, and the high motivation to participate in the assessment, underscores the significance of our sample. The possibility that respondents under-reported actual drug consumption would suggest that the problem is more severe than the data indicate. Another point of criticism might be the quantitative assessment of khat consumption. Numerous qualities of khat with different contents of psychoactive agents are known [71] and studies have shown that the “miraa”-type khat, which is consumed in southern Somalia, probably has a higher cathinone content [72]. But there is reason to believe that there is a positive relationship between the consumption of traded units and consequences, because in regions where higher numbers of khat bundles were consumed, we also found a higher level of sleepless nights due to khat use—a very common consequence; thus, with this kind of definition of consumed quantity we used a rather conservative estimation method. We argue, as have other researchers [68,73], that the assessment of traded units is a viable compromise in the absence of other quantitative methods applicable under present field conditions.

Future research should target the changes in patterns of drug intake in Somalia, determine the actual prevalence and consequences of the consumption of the various drugs, and investigate the changes of underlying regulative social norms and protective mechanisms in society, such as the traditional knowledge of the dangers of khat. We fear that the alarmingly high increase of drug intake in other countries of Africa [25] find their parallel among active Somali fighters, as well as among other especially vulnerable persons, and have already produced a drug problem of a formerly unknown magnitude and quality in the Horn of Africa—at least in some geographical parts and social groups. A future demobilization program should be prepared to meet this challenge [8]. The fact that many of the unit members are former child soldiers and have probably been exposed to combat and drugs during adolescence warrants attention, because they might be especially affected by PTSD and other psychiatric disorders [44]. In the field of addiction treatment, much research has been done in Western countries, but it is unclear to what extent these measures and therapeutic programs can be transferred to settings like Somalia [74]. Rather, research has to be undertaken in order to develop adequate tools, incorporating the relevant cultural and religious aspects as well as the mental health status in the population. Khat prohibition, as it was attempted in the past [75], does not seem to be a feasible solution as recreational khat use is deeply rooted in the local culture. To address this problem, we suggest strengthening both regulation mechanisms inherent to the Somali culture and consumer awareness of the dangers of drug abuse to health.

## Supporting Information

Alternative Language Abstract S1 Translation of Abstract into German (26 KB DOC)

Alternative Language Abstract S2 Translation of Abstract into Italian (22 KB DOC)

Alternative Language Abstract S3 Translation of Abstract into French (23 KB DOC)

## Abbreviations

CI: confidence interval
DDR: disarmament, demobilization, and reintegration
NGO: nongovernmental organization
PTSD: post-traumatic stress disorder
SD: standard deviation
SE: standard error
TFG: Transitional Federal Government
UIC: United Islamic Courts
