# Supplementary material for: The Consumption of Khat and Other Drugs in Somali Combatants: A Cross-Sectional Study
Source: PLoS Med. 2007 Dec 11;4(12):e341. doi: 10.1371/journal.pmed.0040341 (PMC2121109; doi:10.1371/journal.pmed.0040341)
Supplement: Supplementary file 1 [file pmed.0040341.sd001.doc]

Zusammenfassung:

Hintergrund: Seit über einem Jahrzehnt steht der größte Teil Somalias unter keinerlei Regierungskontrolle. Dieser ‚Failure of State’ ist am deutlichsten im Zentrum und Süden des Landes und wird am deutlichsten in der Hauptstadt Mogadishu, die während der langer Jahre von sich gegenseitig bekämpfenden ‚Warlords’ und ihren Milizen kontrolliert wurde. Im Gegensatz dazu wurde der Norden des Landes durch Regionalregierungen stabilisiert, die jedoch keine internationale Anerkennung haben. Die vorliegende Studie über Drogenkonsum konzentriert sich auf aktive Angehörige regulärer bewaffneter Einheiten und Milizen und hebt regionale Unterschiede im Bezug auf den Einfluss staatlicher Ordnung hervor – nach unserer Erkenntnis ist dies die bislang erste derartige Studie.

Methoden/Hauptergebnisse: Trainierte einheimische Interviewer führten insgesamt 8,723 Interviews mit Angehörigen regulärer bewaffneter Einheiten und Milizen in sieben unsystematisch gewonnenen Stichproben in verschiedenen Regionen Somalias durch; 587 (6.3%) Personen brachen das Interview ab und 12 wurden aus anderen Gründen von der Auswertung ausgeschlossen. Neben grundlegenden soziodemographischen Informationen wurden der selbstberichtete Khatkonsum und der in den jeweiligen Einheiten wahrgenommene Konsum von Khat, Cannabis, Psychopharmaka (z.B. Benzodiazepine), Alkohol, Lösungsmittel und Hanfsamen erhoben. Auch bei vorsichtiger Interpretation sprechen die Daten dafür, dass sich bewaffnetes Personal hinsichtlich soziodemographischer Charakteristika und Drogenkonsum erheblich zwischen dem Norden Somalias und den südlichen bzw. zentralen Landesteilen unterscheidet. Insgesamt geben 36.4% der Befragten an, in der Woche vor dem Interview Khat konsumiert zu haben (KI 99% 19.3 – 57.7%), wobei in einigen südlichen und zentralen Landesteilen Khatkonsum an sich und vor allem exzessiver Konsum häufiger berichtet wird. Angaben zum eigenen Khatkonsum und die Wahrnehmung des Konsums in den eigenen militärischen Einheiten unterschieden sich deutlich. In der Wahrnehmung der Befragten ist Khat die am häufigsten konsumierte Droge (im Durchschnitt von 70,1% in der vorangegangenen Woche, KI99% 63.6 – 76.5%), gefolgt von Cannabis (10.7%, KI99% 0 – 30.4%), Psychopharmaka (8.5%, KI99% 0 – 24.4%), Alkohol (5.3%, KI99% 0 – 13.8%), Lösungsmittel (1.8%, KI99% 0 – 5.1%) und Hanfsamen (0.6%, KI99% 0 – 2.0%). Der wahrgenommene Khatkonsum in den eigenen Einheiten unterscheidet sich kaum zwischen Nord- und Südsomalia, während der wahrgenommene Konsum aller anderen Drogen alarmierend hohe Werte in einigen Regionen Südsomalias erreicht, besonders im Bezug auf Cannabis und Psychopharmaka.

Schlussfolgerungen/Relevanz: Die Daten legen nahe, dass sich der Konsum von Drogen während der lang dauernden Konflikte in Südsomalia quantitativ und qualitativ verändert hat, vor allem weil die hier dokumentierten Konsummustern erheblich von den traditionellen abweichen. Unsere Ergebnisse müssen zwar in Studien mit Zufallsstichproben repliziert werden, aber wir ziehen dennoch schon jetzt den Schluss, dass in Südsomalia die Drogenprobleme in bewaffneten Einheiten und anderen Risikogruppen bislang unbekannte Dimensionen erreicht haben, besonders weil wir vermuten, dass mit unserer Erhebungsmethode der wahre Drogenkonsum unterschätzt wird. Wir empfehlen, dass zukünftige Demobilisierungs-, Entwaffnungs- und Reintegrationsprogramme in Somalia auf ein erhebliches Drogenproblem vorbereitet sind.
