# Supplementary material for: The Consumption of Khat and Other Drugs in Somali Combatants: A Cross-Sectional Study
Source: PLoS Med. 2007 Dec 11;4(12):e341. doi: 10.1371/journal.pmed.0040341 (PMC2121109; doi:10.1371/journal.pmed.0040341)
Supplement: Supplementary file 2 [file pmed.0040341.sd002.doc]

Abstract:

Contesto: da oltre dieci anni la Somalia non è controllata da alcun governo. Questo “failure of state” è completo nelle regioni centrali e meridionali e più evidente in Mogadiscio, che è stata nelle mani dei signori della guerra che hanno dispiegato le loro milizie private in una battaglia per le risorse. Al contrario, la parte nord della Somalia è stata sotto il controllo relativamente stabile delle amministrazioni regionali, che non sono tuttavia riconosciute al livello internazionale.

Il presente studio fornisce informazioni circa l’uso di droghe tra il personale di sicurezza attivo e le milizie, con un’enfasi sulle differenze regionali in relazione alla mancanza di controllo di un governo centrale – secondo le nostre informazioni causa primaria di questo fenomeno.

Metodo/Risultati principali: Intervistatori locali addestrati hanno condotto totalmente 8.723 interviste al personale armato, in sette campioni non-random in differenti regioni della Somalia; 587 (6.3%) hanno fornito interviste incomplete e 12 sono stati esclusi per altri motivi. Sono state valutate le informazioni socio-demografiche di base, l’uso auto-riferito di khat e come veniva percepito dai partecipanti l’uso di khat, marijuana, pastiglie psicoattive (come benzodiazepine), alcohol, solventi e semi di canapa all’interno della loro unità. Una interpretazione ponderata dei nostri dati indica che le caratteristiche socio-demografiche e l’uso di droga del personale militare differisce sostanzialmente tra la Somalia settentrionale e quella centro\meridionale. In totale, il 36.4% (IC 99% 19.3 – 57.7%) degli intervistati ha riferito di avere fatto uso di khat nella settimana precedente all’intervista. Dalla stessa intervista però emerge che in alcune regioni della Somalia centro\meridionale viene auto-riferito più frequentemente un uso generale di khat e soprattutto viene riferito un uso eccessivo più frequente. L’uso di khat auto-riferito differiva profondamente dall’uso che veniva percepito nelle unità. Secondo la percezione degli intervistati, la gomma di khat è il tipo di droga usato più spesso (in media, il 70.1% nella settimana precedente, IC 99% 63.6 – 76.5), seguito dal fumo di hashish (10.7%, IC 99% 0 – 30.4), dall’ingestione di pastiglie psicoattive (8.5%, IC 99% 0 – 24.4), dall’alcohol (5.3, IC 99% 0 – 13.8%), dall’inalazione di solventi (1.8%, IC 99% 0 – 5.1) ed infine dall’ingestione di semi di canapa (0.6%, IC 99% 0 – 2.0). L’uso di khat percepito è leggermente diverso tra nord e sud della Somalia, mentre l’uso percepito di altre droghe raggiunge livelli allarmanti in alcune regioni del sud specie in relazione all’uso di cannabis e pastiglie psicoattive.

Conclusioni\Rilevanza: I nostri dati suggeriscono che l’uso di droghe è cambiato sia dal punto di vista quantitativo che qualitativo nel corso dei conflitti nel sud della Somalia, poiché l’uso corrente è diverso dall’uso tradizionale. Sebbene siano necessari ulteriori studi che usano un metodo di campionamento random per confermare i nostri dati, concludiamo che i problemi legati alla droga nel personale armato ed in altri gruppi vulnerabili nel sud della Somalia può avere raggiunto proporzioni fino ad oggi sconosciute nel Paese se si considera anche che, a nostro parere, il bias del nostro studio tende verso una sottostima del fenomeno reale. Noi auspichiamo quindi che i futuri programmi di disarmo, smobilitazione e reintegrazione (DDR) siano preparati ad far fronte ai sostanziali problemi relativi alla droga in Somalia.
