# Supplementary material for: The Consumption of Khat and Other Drugs in Somali Combatants: A Cross-Sectional Study
Source: PLoS Med. 2007 Dec 11;4(12):e341. doi: 10.1371/journal.pmed.0040341 (PMC2121109; doi:10.1371/journal.pmed.0040341)
Supplement: Supplementary file 3 [file pmed.0040341.sd003.doc]

Contexte :

Cela fait maintenant plus de dix ans que la majorité des régions de Somalie ne sont contrôlées par aucune sorte de gouvernement. Cet ‘échec de l’Etat’ est total dans les régions du centre et du sud, et particulièrement apparent à Mogadiscio, qui fut pendant une longue période aux mains de chefs de guerre déployant leurs milices privées dans une lutte pour les ressources. En revanche, la partie nord de la Somalie a connu un contrôle relativement stable par des administrations régionales, qui ne sont cependant pas reconnues internationalement. Cette étude vise à fournir des informations sur l’usage de drogues parmi le personnel de sécurité actif et les membres de la milice, avec un accent sur les différences régionales liées au manque de contrôle par un gouvernement central – c’est à notre connaissance la première étude sur ce sujet.

Méthodologie / Résultats principaux :

Des enquêteurs locaux entraînés ont mené un total de 8723 interviews de personnel armé sur sept échantillons non randomisés dans différentes régions de la Somalie ; 587 (6.3%) des personnes interrogées ont arrêté l’interview en cours et 12 ont été exclus pour d’autres raisons. Nous avons évalué les données socio-économiques de base, l’usage personnel de khat et la manière dont les personnes interrogées percevaient au sein de leurs unités l’utilisation du khat, de la marijuana, des médicaments psycho-actifs (ex. : benzodiazépines), de l’alcool, des solvants et du chènevis (graine de chanvre indien). L’interprétation menée avec prudence de nos données suggère que les caractéristiques socio-démographiques et l’usage de drogues par le personnel militaire diffèrent sensiblement entre les régions du nord de la Somalie et celles du sud/centre. Au total, 36.4% (IC99% 19.3 – 57.7%) des personnes interrogées ont déclaré avoir utilisé du khat dans la semaine précédent l’interview, alors que dans certaines régions de la Somalie centrale et du sud l’usage du khat en général, et plus spécifiquement l’usage excessif, a été rapporté comme étant plus fréquent. L’usage personnel de khat était significativement différent de celui perçu au sein des unités. D’après la perception des personnes interrogées, la mastication des feuilles de khat est la drogue la plus fréquemment consommée (en moyenne, 70.1% la semaine précédente, IC99% 63.6 – 76.5), suivi par le haschich fumé (10.7%, IC99% 0 – 30.4), l’ingestion de médicaments psycho-actifs (8.5%, IC99% 0 – 24.4), l’ingestion d’alcool (5.3, IC99% 0 – 13.8%), l’inhalation de solvants (1.8%, IC99% 0 – 5.1) et l’absorption de chènevis (0.6%, IC99% 0 – 2.0). La perception de l’usage du khat diffère peu entre le nord et le sud de la Somalie, mais la perception de l’usage d’autres drogues atteint des niveaux élevés alarmants dans certaines régions du sud, en particulier pour la consommation de cannabis fumé et de médicaments psycho-actifs.

Conclusions :

Nos données suggèrent que l’usage de drogues a quantitativement et qualitativement changé au cours des conflits dans la partie sud de la Somalie, les schémas actuels contrastant avec l’usage traditionnel. Bien que de futures études utilisant des méthodes d’échantillonnage randomisé doivent encore confirmer nos résultats, nous concluons que les problèmes liés à la drogue du personnel armé et des autres groupes vulnérables dans la partie sud de la Somalie, ont probablement atteint des proportions précédemment inconnues dans ce pays. Ce sentiment est d’autant plus fort que nous croyons que des biais systématiques dans nos données devraient mener à une sous-estimation de l’usage réel de drogue. Nous recommandons que de futurs programmes de désarmement, de démobilisation et de réintégration (DDR) soient préparés pour gérer des problèmes significatifs liés à la drogue en Somalie.
